# Antimicrobial Efficacy of a Vegetable Oil Plasticizer in PVC Matrices

**DOI:** 10.3390/polym16081046

**Published:** 2024-04-10

**Authors:** Greta Bajetto, Sara Scutera, Francesca Menotti, Giuliana Banche, Giuseppe Chiaradia, Caterina Turesso, Marco De Andrea, Marta Vallino, Daan S. Van Es, Matteo Biolatti, Valentina Dell’Oste, Tiziana Musso

**Affiliations:** 1 Department of Public Health and Pediatric Sciences, University of Turin, 10100 Turin, Italy; greta.bajetto@unito.it (G.B.); sara.scutera@unito.it (S.S.); francesca.menotti@unito.it (F.M.); giuliana.banche@unito.it (G.B.); marco.deandrea@unito.it (M.D.A.); valentina.delloste@unito.it (V.D.); tiziana.musso@unito.it (T.M.); 2 Center for Translational Research on Autoimmune and Allergic Disease—CAAD, 28100 Novara, Italy; 3 Fluos Sas, 21052 Busto Arsizio, Italy; chiaradia@fluos.it (G.C.); turesso@fluos.it (C.T.); 4 Institute for Sustainable Plant Protection, National Research Centre (CNR), 10135 Turin, Italy; marta.vallino@ipsp.cnr.it; 5 Wageningen Food & Biobased Research, 6708 WG Wageningen, The Netherlands; daan.vanes@wur.nl

**Keywords:** plasticizers, biomaterials, vegetable oils, polyvinyl chloride (PVC), antiviral and antibacterial polymers, medical devices

## Abstract

The growing prevalence of bacterial and viral infections, highlighted by the recent COVID-19 pandemic, urgently calls for new antimicrobial strategies. To this end, we have synthesized and characterized a novel fatty acid epoxy-ester plasticizer for polymers, named GDE. GDE is not only sustainable and user-friendly but also demonstrates superior plasticizing properties, while its epoxy components improve the heat stability of PVC-based matrices. A key feature of GDE is its ability to confer antimicrobial properties to surfaces. Indeed, upon contact, this material can effectively kill enveloped viruses, such as herpes simplex virus type 1 (HSV-1) and the β-coronavirus prototype HCoV-OC43, but it is ineffective against nonenveloped viruses like human adenovirus (HAdV). Further analysis using transmission electron microscopy (TEM) on HSV-1 virions exposed to GDE showed significant structural damage, indicating that GDE can interfere with the viral envelope, potentially causing leakage. Moreover, GDE demonstrates antibacterial activity, albeit to a lesser extent, against notorious pathogens such as *Staphylococcus aureus* and *Escherichia coli*. Overall, this newly developed plasticizer shows significant potential as an antimicrobial agent suitable for use in both community and healthcare settings to curb the spread of infections caused by microorganisms contaminating physical surfaces.

## 1. Introduction

Surface contamination is increasingly being recognized as a significant contributor to the spread of both notorious community-acquired and healthcare-associated infections (HAIs) [1]. Indeed, nearly 80% of human infections are derived from surfaces contaminated with microbes [2]. Given that humans spend most of their time indoors [3], where they are in continuous contact with objects that may harbor microbial contaminants, preventing the colonization of pathogens on physical surfaces has become a critical aspect of infection control.

Despite the increasing use of biomaterials in everyday life, there is still a lack of specifically engineered materials designed to effectively mitigate the occurrence of infections. Current efforts involve the application of various types of antimicrobial coatings on surfaces, including antibiotics, antimicrobial peptides, and quaternary ammonium compounds [4,5]. However, these approaches are often limited by the induction of resistance or their short-term effectiveness. Furthermore, even though biocidal surfaces may help reduce pathogen spread, their efficacy can also be affected by evaporation or removal, requiring frequent disinfection treatments, especially in high-risk areas, such as hospitals, public buildings, and transport systems. Thus, there is growing interest in developing new polymer-based formulations to counter bacterial resistance development by eradicating pathogens through a process that disrupts and bursts their membranes [6]. A major challenge with these surfaces is the buildup of dead microbes on the coatings, which can block the antimicrobial functional groups, significantly reducing their effectiveness. For instance, Hancock SN et al. conducted a study wherein they synthesized novel cationic polymers through controlled ring-opening metathesis polymerization of N-methylpyridinium-fused norbornenes. These polymers exhibited selective activity against both Gram-positive and Gram-negative bacteria, while sparing human red blood cells [7]. Furthermore, research has explored the utility of antimicrobial polymers such as chitosan [8,9] and polyethyleneimine [10], demonstrating promising outcomes in rupturing bacterial cell membranes, resulting in bacterial demise. Additionally, investigations have delved into polymer-based nanoparticles loaded with antimicrobial agents, showcasing different mechanisms of action [11,12,13].

Polyvinyl chloride (PVC) stands as a prevalent thermoplastic polymer in commercial applications and ranks among the most common surfaces utilized within the medical sector. Ongoing research endeavors focus on the modification of PVC to imbue it with antimicrobial properties. Such enhancements hold promise in mitigating the risk of infections and cross-contamination across various industrial processes [14,15].

Plasticizers are commonly added to the PVC resin to impart flexibility and thermal stability. Among the main PVC plasticizers are phthalates, including di(2-ethylhexyl) phthalate (DEHP) and diisononyl phthalate (DINP), as well as adipates, trimellitates, and epoxidized soybean oil. These plasticizers enhance the workability of PVC materials, making them suitable for diverse applications, spanning from construction to automotive and healthcare [16]. However, despite their utility, PVC plasticizers have drawn scrutiny due to potential health and environmental concerns. Phthalates, in particular, have been associated with adverse health effects, including endocrine disruption and reproductive toxicity, raising regulatory and consumer awareness. Additionally, there are concerns about their leaching potential, leading to the contamination of the environment and potential human exposure [17,18,19]. Despite the increasing recognition of the adverse effects associated with phthalates, particularly DEHP, it remains the predominant plasticizer worldwide and is often present in commercial PVC at concentrations ranging from 30 to 40% wt [20]. To address concerns regarding the usage of DEHP and similar phthalate plasticizers, recent research has shifted toward the development of bio-based alternatives that offer comparable performance without compromising the properties of the final product [21,22].

Against this background, we have developed and characterized a novel bio-based fatty acid derivative, named GDE, which acts as a plasticizer within a PVC matrix. In particular, we have evaluated this compound for various properties and compared it to a set of relevant commercial benchmarks, both in its pure form and as part of flexible PVC formulations. Finally, we have tested the antimicrobial efficacy of GDE-infused matrices against several viruses (i.e., herpes simplex virus type 1 (HSV-1), the β-coronavirus HCoV-OC43, and human adenovirus (HAdV)) and two human pathogenic bacteria, the Gram-positive *Staphylococcus aureus* and the Gram-negative *Escherichia coli*.

The implications of using GDE in healthcare settings, focusing on its potential role in enhancing infection control measures, will be discussed below.

## 2. Materials and Methods

### 2.1. Materials

The materials employed in this study are reported in Table 1.

### 2.2. Analysis of the Chemical and Physical Properties of the Plasticizer

*Ester content*. The ester content was determined by gas chromatography-mass spectrometry (GC-MS) analysis using an Interscience Trace GC Ultra GC + PTV with AS3000 II autosampler (Conquer Scientific, Poway, CA, USA).

*Acid values.* The acid values were determined by colorimetric titration according to the BS EN ISO 2114 2000 standard [24].

*Water content.* The water content was determined using Karl-Fischer titration, as previously reported [25].

*Static viscosity.* The static viscosity was measured using an ARES-G2 rheometer (TA Instruments, New Castle, DE, USA) equipped with a forced convection oven (−150 °C to 600 °C) and a two-step cooling unit. The data analysis was performed with Trios software version 5.2 (TA Instruments, New Castle, DE, USA).

*Thermo-gravimetric analysis.* The thermo-gravimetric analysis (TGA) analyses were performed on a Perkin Elmer STA 6000 (simultaneous thermal analyzer, PerkinElmer, Waltham, MA, USA) from 30–900 °C at 10 °C/min under an N_2_ flow (30 mL/min) for 15 min After 15 min at 900 °C, the gas flow was switched to air (30 mL/min). The results (weight loss) were evaluated with Perkin Elmer Pyris software version 11 (PerkinElmer, Waltham, MA, USA).

*Color measurements.* The color measurements were performed using a Konica Minolta CM-5 bench-top spectrophotometer (Konica Minolta, Tokyo, Japan), and the values were expressed according to the American Public Health Association (APHA) scale.

### 2.3. PVC Property Methods

*Preparation of plasticized PVC test samples.* To prepare the PVC formulations, PVC resin, at a ratio of 100 parts per hundred resin (phr), primary and secondary thermal stabilizers (Ba-Zn and ESBO), and two concentrations (40 or 60 phr) of various plasticizers (i.e., GDE, DINP, DINCH, DEHTP, G-SNS, and P-401) were mixed at 2000 rpm for 1.5 min using a SpeedMixer™ DAC 150 FVZ (FlackTek, Louisville, CO, USA). The detailed composition of each formulation is shown in Table 2. Subsequently, these formulations were processed at 166 °C for 5 min on a two-roll mill (Collin Type 110P, Collin Lab & Pilot Solutions, Maitenbeth, Germany) and then compression molded into 1 mm thin sheets at 170 °C under a pressure of 37 kg/cm^2^. The processed sheets were immediately cooled, and samples were cut out for further testing.

#### 2.3.1. Thermal Properties

*Static heat stability test.* Static heat stability (SHS) tests were performed on the plasticized PVC sheets, which were prepared using a two-roll mill, with a Thermotester LTE-T (Werner Mathis AG, Oberhasli, Switzerland). The PVC films, cut to dimensions of 250 × 15 mm, were placed in the oven, with a ventilation speed of 2000 rpm at a temperature of 200 °C. The oven heat constancy test was conducted over a total residence time of 83 min, with the program set to move the tray out of the oven at intervals of 2 mm/40 s.

*Congo red test.* Congo red (CR) experiments were conducted according to the ISO 182-1:1992 standard [26]. The temperature-controlled oil bath was set at 180 °C. The samples were placed into a closed test tube and submerged in the oil bath until the CR paper strip transitioned from red to blue. The time required for this chromatic transition, known as the stability time, is defined as the minutes elapsed from the start (t = 0) to the color change. The epoxy plasticizer P-401 was used as a control.

#### 2.3.2. Mechanical Properties

*Tensile testing.* The mechanical properties, including the ultimate tensile strength (E-modulus), breaking strength (F-max), and maximum elongation (strain at fracture expressed as %), were measured with a Universal Testing Machine Z010 AllroundLine (Zwick/Roell, Venlo, The Netherland) using a 1 kN load cell with a crosshead speed of 1.0 mm/min or 500 mm/min for the E-modulus and F-max and maximum elongation measurements, respectively, in accordance with ISO-37:2017 [27] and ISO-1184:1983 [28].

*Dynamic mechanical thermal analysis.* The dynamic mechanical thermal analysis (DMTA) of the PVC films was performed using a Rheometrics Solids Analyzer (RSA) II (TA Instruments, New Castle, DE, USA) equipped with a film geometry. The testing was performed at a frequency of 1 Hz, with a heating rate of 3 °C/min, ranging from −60 to 80 °C. The glass transition temperature (Tg)—the point at which the material changes from a glassy to a soft state—was identified at the peak of the tanδ curve.

*Shore A measurements.* The Shore A hardness of the PVC samples was measured using a hardness tester (Zwick/Roell, Genova, Italy), in compliance with the ISO 868:2003 standard guidelines [29].

### 2.4. Antimicrobial Assay

*Preparation of PVC matrices plasticized with GDE and control DEHTP.* To prepare the PVC matrices, PVC resin, thermal stabilizers (Ba-Zn and ESBO), and either GDE or DEHTP plasticizers were used at different concentrations. With respect to the PVC resin (100 parts), Ba-Zn and ESBO were used at 1 phr and 5 phr, respectively, across all the formulations. The plasticizer GDE was incorporated at varying concentrations by weight (wt): 50% wt (106 phr), 36% wt (60 phr), 23% wt (32 phr), or 10% wt (12 phr). For comparison, each test includes DEHTP at its highest concentration of 50% wt. DEHTP served as the benchmark plasticizer, chosen for its established safety profile, which makes it a safer alternative to DEHP in childcare and medical devices [30]. All the components were blended in a turbo-mix at 80 °C for 30 min. Successively, the mixture was processed through a roll calendar to produce sheets of flexible PVC.

#### 2.4.1. Antiviral Assays

*Viral strains and cell lines.* For the virus studies, we used the human adenovirus type 5 (HAdV, purchased from the “American Type Culture Collection, ATCC”, VR-5), a clinical isolate of human herpes simplex virus type 1 (HSV-1), kindly provided by Dr. Valeria Ghisetti of the “Amedeo di Savoia” Hospital, Turin, Italy, and the human coronavirus strain OC43 (HCoV-OC43), (ATCC VR-1558), a gift from Prof. David Lembo, Department of Clinical and Biological Sciences, University of Turin, Turin, Italy. The HSV-1 and HAdV were propagated and titrated by plaque assay on African green monkey kidney cells (VERO, ATCC CCL-81), whereas the HCoV-OC43 was handled using human lung fibroblast cells (MRC-5, ATCC CCL-171), as previously described [31,32]. The cells were cultured in Dulbecco’s Modified Eagle’s Medium, supplemented with 10% heat-inactivated fetal bovine serum (FBS), 2 mM glutamine, 1 mM sodium pyruvate, 100 U/mL penicillin, and 100 µg/mL streptomycin sulfate (Sigma-Aldrich, Milan, Italy).

*Preparation of sample surfaces.* To remove any contaminants that could interfere with the analysis of the antiviral activity, the PVC-based matrices underwent UV exposure for 20 min on each side. The matrices were then placed in a polystyrene petri dish, to which we added a fixed amount of virus inoculum, diluted in 500 µL of the complete growth medium. This mixture was centrifuged at 2000 rpm for 15 min to increase the virus adsorption and then incubated at different time points at room temperature. Subsequently, the samples were collected, and the residual viral infectivity was determined by standard plaque assay (see below). The infectious viral titers obtained from these experiments were compared to those from the benchmark plasticizer DEHTP and a control virus not exposed to the matrices to determine the relative reduction in the viral titer after contact with the matrices.

*Plaque assay.* The HSV-1, HAdV, and HCoV-OC43 titers were assessed by the standard plaque method on fully confluent VERO or MRC-5 cells in 96-well plates. Each well was inoculated with 10-fold serial dilutions of the sample. After 48 h, the plates were fixed and stained with a 0.1% crystal violet solution (Sigma-Aldrich, Milan, Italy). The cytopathic effect of HSV-1 and HAdV was scored by observation under an inverted microscope. The virus titers (plaque-forming unit per milliliter, PFU/mL) were calculated by counting the number of foci on each well using the following formula: number of plaques ×0.1 mL/dilution factor. For HCoV-OC43, infectivity was determined by measuring the absorbance at 595 nm using a Victor X4 multilabel plate reader (PerkinElmer, Waltham, MA, USA). Histograms were obtained by plotting the absorbance values at the 10^−4^ dilution, as a percentage of the mean absorbance of the uninfected control (mock) (set as 100%), for each compound concentration.

*Transmission electron microscopy.* For HSV-1, the preparations were applied to the GDE matrices or DEHTP controls, as described above, maintained for 5 h, and then removed by gentle pipetting. The samples were adsorbed onto carbon- and PELCO^®^ Formvar-coated copper grids (TED PELLA, Redding, CA, USA), left to stand for 5 min, washed with water, and then negatively stained using 0.5% aqueous uranyl acetate. Images were captured using a CM10 electron microscope (Philips, Amsterdam, The Netherlands) operating at 60 kV. The counting of intact or damaged virions in each grid hole was performed during observation.

#### 2.4.2. Antibacterial Assays

*Bacterial strains.* Antibacterial assays were performed on *S. aureus* (ATCC 29213) and *E. coli* (ATCC 25922), serving as representative Gram-positive and Gram-negative bacteria, respectively. The two bacterial strains were cultured on Mannitol Salt Agar (MSA, Oxoid, Rodano, Italy) for *S. aureus* and MacConkey Agar (MAC, Oxoid, Rodano, Italy) for *E. coli*. Young colonies (18–24 h old) were stored at −80 °C.

*Quantification assays of adherent and planktonic bacteria.* To evaluate the adhered and planktonic cells on the treated surfaces, we followed the method previously reported by Cazzola M et al. [33]. Briefly, different PVC-based disks were sterilized under UV for 20 min on each side, transferred into a 6 multi-well sterile culture plate, and covered with a bacterial suspension (1 × 10^4^ CFU/mL) of either *S. aureus* or *E. coli* in Mueller Hinton Broth (MHB, Sigma-Aldrich, Milan, Italy) medium. The plates were centrifuged at 2000 rpm for 10 min to allow bacterial adhesion to the PVC matrices and then incubated at 37 °C for 5 h. The controls involved bacteria of the same inoculum size incubated in MHB with no material. The total bacteria count, comprising those firmly attached to the matrices and planktonic cells, was quantified by CFU/mL count on Mueller Hinton Agar (MHA, Sigma-Aldrich, Milan, Italy). Specifically, the bacteria adhered to the PVC samples were detached from each disk using a sonication protocol at 40 kHz for 10 min at 22 °C in sterile 0.9% NaCl and then quantified. On the other hand, the planktonic bacteria were assessed by directly plating 100 µL of the bacterial suspension directly onto agar plates.

*Inhibition halo test.* The potential release of substances with antibacterial properties from the same matrices was evaluated through an inhibition halo assay against the same bacterial strains. In detail, disks of the DEHTP 50% and GDE matrices at different percentages (50%, 36%, 23%, or 10%) were cut into 6 mm diameter disks and placed on MHA plates, which had been pre-inoculated with a 0.5 McFarland bacterial suspension. Standard gentamicin discs (10 µg/disc) were used as a positive control for antimicrobial activity. After overnight incubation at 37 °C, the antibacterial activity was assessed by measuring the inhibition halo zone (in mm) around the samples.

### 2.5. Statistical Analysis

All the statistical analyses were performed using GraphPad Prism software version 7.00 for Windows (GraphPad, Boston, MA, USA). The data were presented as the mean value ± standard error of the mean (SEM). Differences were considered statistically significant at *p* < 0.05, with * *p* < 0.05, ** *p* < 0.01, and *** *p* < 0.001 indicating increasing levels of significance. For the PVC properties, the data presented were recorded in accordance with the indicated ISO norms.

## 3. Results

### 3.1. Synthesis and Characterization of the Fatty Acid Epoxy-Ester Plasticizer GDE

In our effort to identify biomaterials with enhanced microbiocidal activity, initial experiments focused on synthesizing fatty acid esters derived from natural vegetable oils. These esters, once combined with cyclic hydroxy acetal and epoxidized in the double bonds of their fatty acid hydrocarbon chains, displayed exceptional plasticizing properties and remarkable compatibility with PVC. Specifically, the fatty acid epoxy-ester glycerol formal referred to as GDE (patent PCT/EP2014/074208) was obtained by transesterification of fatty acid methyl esters with glycerol formal and subsequent epoxidation of the double bonds using aqueous hydrogen peroxide and formic acid solution. The fatty acids used in this reaction were chosen from those with hydrocarbon chains between C12 and C22, where the double bonds of the hydrocarbon chain of the carboxylic fatty acid are epoxidized.

**Figure 1:**
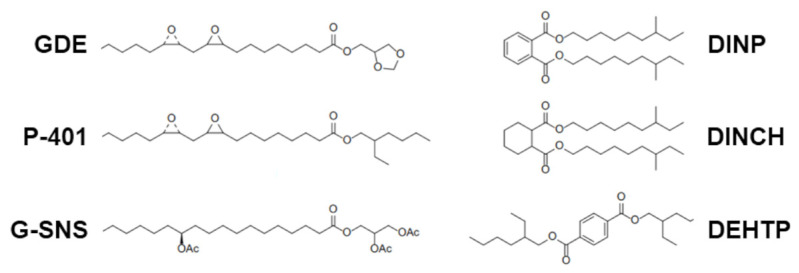
Molecular structure of the fatty acid epoxidized ester (GDE) with oleic acid and benchmarks used for comparison in this study.

The main chemical and physical properties of GDE were determined and then compared with representative benchmark plasticizers, DINP, DINCH, DEHTP, G-SNS, and P-401 (Figure 1, Table 1), as summarized in Table 3.

The ester content of the plasticizers was quantified by GC-MS analysis (Figure S1). Based on the chosen criteria for peak selection—whether taking into account only the main peaks or including intermediated peaks as well—the ester content was determined to be either 95.7% or 98.4%, respectively. This occurrence of multiple peaks is consistent with the fact that GDE is a composite derived from various compounds. This complexity results from a number of factors, such as the type of the vegetable oil feedstock used, the number of unsaturations, the extent of the epoxidation, and the isomeric distribution present in the glycerol formal.

Consistent with the characteristics of vegetable oil-derived products, the water content of GDE is extremely low, similar to that observed in the benchmark controls. The acidity of the GDE product is higher than that of phthalates, but it is similar to that of other epoxidized soybean esters, such as ESBO. The viscosity of GDE is comparable to that of G-SNS, a vegetable oil-based product, yet higher compared to all the other benchmarks, including P-401. Both GDE and the control substances are characterized by low volatile and ash content, as determined by TGA analysis. Nonetheless, the color intensity of the GDE product consistently exceeds that of all the other benchmarks, albeit it aligns with the standard specifications for ESBO, which normally displays a color intensity < 200 APHA.

### 3.2. Plasticized PVC Processing and Characterization

The next step in this study was to evaluate the impact of GDE on PVC lamination relative to standard benchmark plasticizers. For this purpose, PVC was mixed with plasticizers at concentrations of 40 and 60 phr along with stabilizers (Table 1). These PVC blends were then processed on a two-roll mill, generating the primary milled sheets of plasticized PVC (Figure S2).

All the samples appeared homogeneous and transparent, with a slightly yellowish color. The transparency of the PVC sheets indicated good compatibility between the plasticizer and the matrix. The color measurements of the roll-milled PVC compounds were performed using their respective press plates. Due to the intrinsic mild yellow tint of properly stabilized PVC, the addition of high amounts (40–60 phr) of a more intensely colored additive like GDE led to a marked increase in the color intensity of the PVC sheets (~210 and 240 APHA for 40 and 60 phr, respectively). However, this intensity was still lower than that of the neat plasticizers, as PVC naturally dilutes the color of the plasticizer. Indeed, the PVC sheets containing low-color plasticizers, such as G-SNS and DINP, at 40 phr exhibited APHA values of 150 and 120, respectively.

### 3.3. Thermal Stability of Plasticized PVC

To evaluate the thermal stability of the PVC formulations, static heat stability (SHS) and Congo red (CR) tests were conducted.

SHS analyses were performed on 40 and 60 phr plasticized PVC sheets using a temperature-controlled aging oven. This analysis aimed to identify any discernible color changes in the PVC samples, which would indicate variations among them.

It is important to point out that this procedure does not measure the absolute thermal stability; rather, it allows a comparative analysis between the GDE plasticizer and the commercial benchmarks under the same conditions.

Three key parameters were assessed during the SHS analysis: (1) early color (i.e., the color of the strip at t = 0 s); (2) color hold (the point at which discoloration becomes evident); and (3) long-term heat stability (the time at which complete degradation starts).

**Figure 2:**
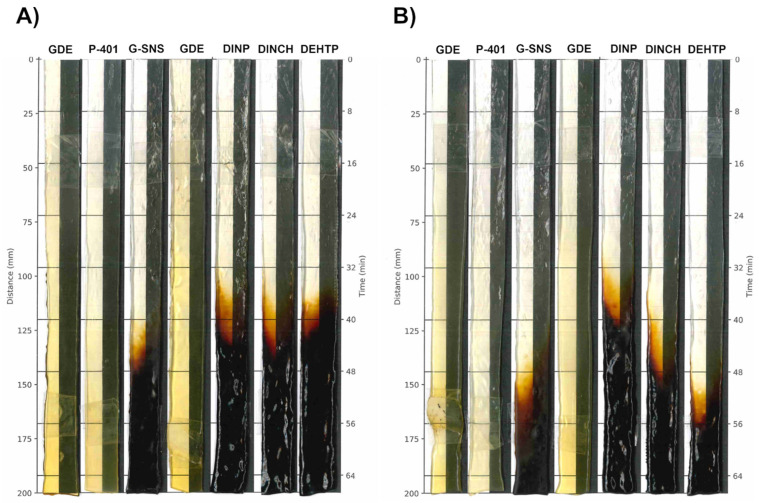
Color changes in PVC formulations with 40 (**A**) or 60 phr (**B**) concentrations. All the sample strips were processed by two-roll mills at 200 °C, followed by automatic removal from the oven at a rate of 2 mm/40 s.

Figure 2 and Table 4 show the effects of discoloration on PVC at 200 °C in air. Differences were observed between the 40 and 60 phr strips of commercial plasticizers (i.e., G-SNS, DINCH, DEHTP), except for DINP. This effect was likely due to the lower percentage of thermally unstable PVC in the compound. Indeed, epoxidized fatty acid derivatives, such as GDE and P-401, significantly enhance long-term heat stability compared to the commercial plasticized PVC used in this study, delaying the rapid degradation of PVC. However, aside from the more pronounced early color of the GDE sheet, which was due to the higher color intensity of the neat plasticizer, the color development during the SHS testing was in line with that observed for P-401, suggesting that the colored components in the GDE samples do not contribute to additional color formation in the PVC sheet during static heat treatment.

To validate the results obtained with the discoloration method, a CR assay was also performed. Since thermal stability usually increases with a higher percentage of plasticizer, the test was specifically performed on the 40 phr formulations. As shown in Table 5, while the DINP compound exhibited an average value of 86 min, based on the clear color change of the indicator paper from red to blue, GDE displayed no color change even after being exposed to temperatures of 180 °C for more than 5 h, indicating the absence of severe degradation.

To ascertain whether the remarkable thermal stability of the GDE PVC compound is a characteristic of epoxidized plasticizers, the experiment was extended to include the P-401 plasticizer. The results shown in Table 5 clearly demonstrate that this epoxy plasticizer confers high thermal stability, as evidenced by the absence of a color change on the CR paper.

### 3.4. Tensile and Mechanical Properties of Plasticized PVC

To investigate the effect of GDE addition on the overall strength of PVC matrices, we measured the tensile strength (E-modulus), breaking strength (F-max), and maximum elongation (strain at break) (Table 6).

Among the samples containing the commercial plasticizers at 40 phr, DINP is known for its high flexibility, as judged by its E-modulus and strain at break values. When comparing the compounds plasticized with fatty acid esters, GDE seemed to confer the highest flexibility. The E-modulus values of the GDE samples were lower compared with those obtained with the bio-based plasticizers P-401 and G-SNS. In contrast, the F-max values obtained with GDE were closer to those found in more traditional plasticizers. Similarly, the strain at break for GDE was also comparable to that of DINP, although there was significant variability in this parameter.

Increasing the plasticizer level to 60 phr resulted in highly flexible materials but with less distinguishing features. The E-modulus values were uniformly low across all the materials tested, with almost no variability. Nevertheless, GDE remained the best-performing plasticizer, showing similar trends to the 40 phr series, albeit less pronounced. A significant difference was seen in the strain at break values, where GDE averaged a value of 336%, far exceeding that of DINP (203%).

All the samples with 40 phr vegetable oil-based plasticizers (i.e., GDE, P-401, and G-SNS) showed similar Shore A hardness values, ranging from 26 to 29, which were lower than those with DINP-, DINCH-, or DEHP-based formulations (range 34–52), suggesting a higher plasticizing efficiency (Table 6).

For the 60 phr series, the differences became less pronounced, which may be due to the fact that the materials reached a softness level that may have exceeded the capabilities of the testing method employed.

The viscoelastic behavior of the different blends was evaluated by DMTA. The analysis of the tanδ curves for the 40 phr formulations showed that GDE lowered the Tg values by more than 10 °C compared to DINP, DINCH, and DEHTP (Figure S3). As a result, materials with low Tg values displayed increased flexibility. For GDE, the Tg was recorded at 20 °C, while for DINP, DINCH, and DEHTP, the Tg values were, respectively, 29, 38, and 32 °C. Among the vegetable oil-based plasticizers, the GDE and G-SNS formulations were characterized by similar Tg values (20 °C and 21 °C, respectively). Despite being structurally analogous to GDE, P-401 was not as effective in lowering the Tg of the blends, with a value of 30 °C.

### 3.5. Antiviral Activity of GDE Matrices

**Figure 3:**
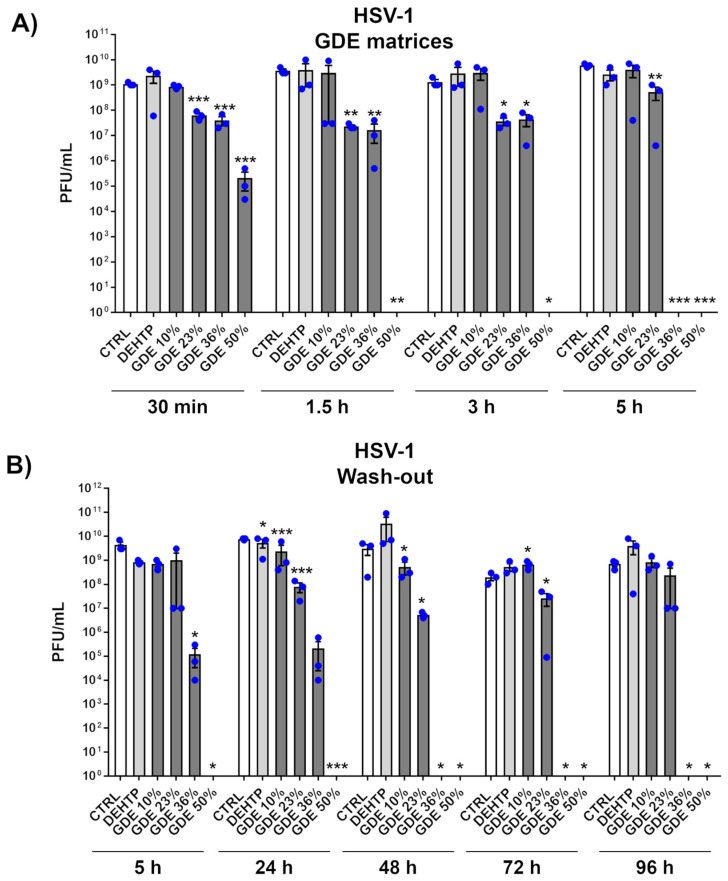
The virucidal activity of the GDE matrices against HSV-1. (**A**) HSV-1 virions (500 µL) were applied to a 1 cm^2^ coupon of PVC matrices containing the indicated percentage of GDE or a benchmark plasticizer (DEHTP 50% wt). An untreated virus sample (CTRL) was used as a positive control. After the indicated contact times, the virus was removed and assayed for infectivity by a standard plaque assay. The plaques were microscopically counted, and the mean plaque counts for each molecule were expressed as plaque-forming unit/mL (PFU/mL). (**B**) The GDE matrices and controls were treated as described in (**A**). At the indicated time points, the viral inoculum was removed and replaced with a fresh inoculum, on the same matrix. The infectivity was determined by a standard plaque assay. Values are expressed as means ± SEM from three independent experiments. Differences were considered statistically significant for *p* < 0.05 (* *p* < 0.05; ** *p* < 0.01; *** *p* < 0.001, unpaired *t*-test, for comparison of GDE or DEHTP vs. CTRL samples).

To determine whether GDE matrices possess virucidal properties, we first selected herpes simplex virus type 1 (HSV-1), a prototype dsDNA and enveloped virus, which is highly transmissible and widespread in the population [34,35]. Next, GDE matrices containing various percentages of GDE plasticizer were incubated with a fixed amount of HSV-1 virions at different time points. The antiviral activity was then assessed through a plaque assay. As depicted in Figure 3A, when exposed to simulated wet-droplet contamination (500 µL per cm^2^) containing approximately 10^9^ PFUs of HSV-1, a strong dose-dependent inactivation was observed after only 30 min, with virtually no virus remaining in the presence of GDE at 36% and 50% concentrations after 5 h. In contrast, matrices containing the DEHTP control exhibited no virucidal activity against HSV-1, indicating that this property is specific to the GDE plasticizer. Of note, the virus suspension was prepared in a complete DMEM growth medium with 10% FBS, which is considered a significant organic protein load. Despite this high protein concentration, the robust virucidal efficacy exhibited by the GDE matrices suggests that their effectiveness may not be affected by the protein content found in human sputum droplets, which are known to shed HSV-1 and other respiratory viruses.

Given that most biocidal substances can evaporate or leach from materials over time, requiring periodic disinfection treatments, we also investigated the reusability and residual virucidal activity of our GDE matrices. In a time-course experiment, the virus was exposed to the same matrix every 24 h for a total of 96 h. As shown in Figure 3B, the GDE matrices maintained their antiviral properties in a dose-dependent manner even after repeated exposures up to 96 h. This durability underscores the enhanced virucidal properties of the GDE formulation, which appears to be capable of neutralizing pathogens after repeated exposures.

**Figure 4:**
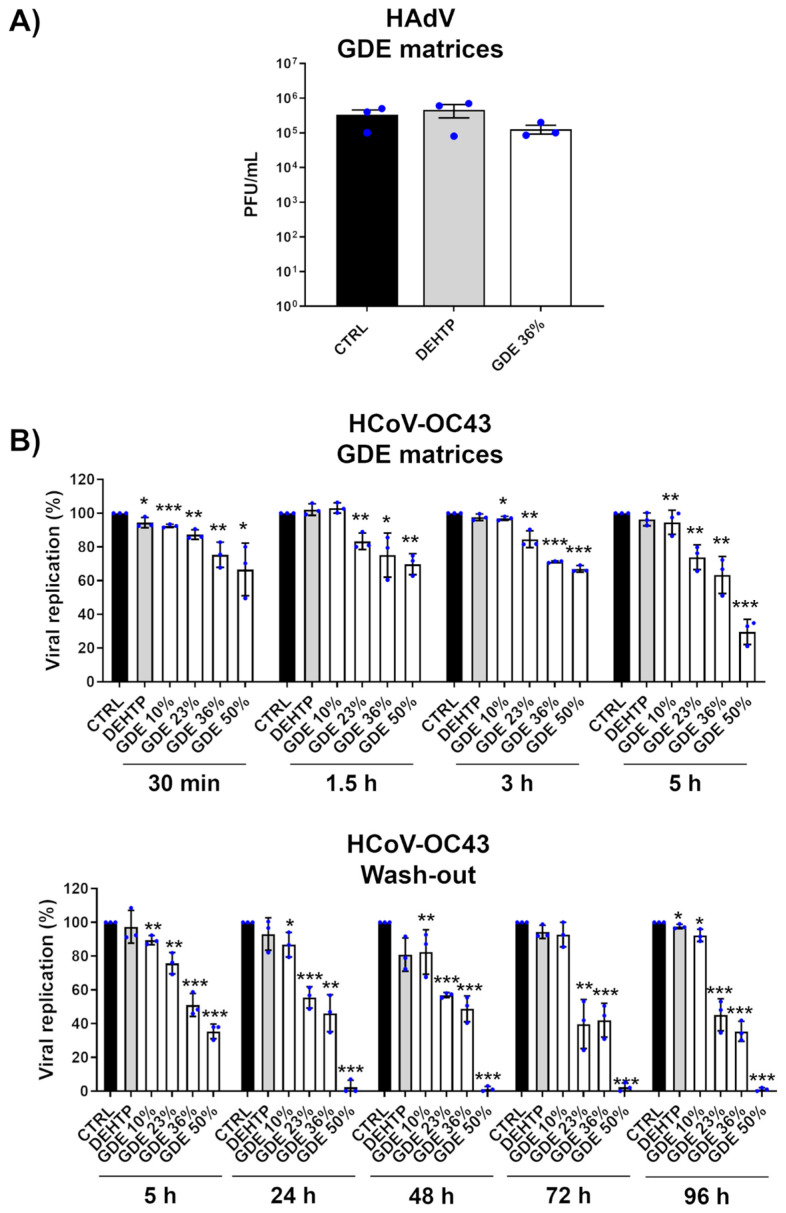
Broad-range virucidal activity of GDE matrices against enveloped viruses. (**A**) HAdV virions (500 µL) were applied to a 1 cm^2^ coupon of PVC matrices containing the indicated percentage of GDE or a benchmark plasticizer (DEHTP 50% wt). An untreated virus sample (CTRL) was used as a positive control. After the indicated contact times, the virus was removed and assayed for infectivity by a standard plaque assay. The plaques were microscopically counted, and the mean plaque counts for each molecule were expressed as plaque-forming unit/mL (PFU/mL). (**B**) (**Upper panel**). The GDE-treated PVC matrices and controls were treated as described in (**A**). At the indicated time points, the viral inoculum was removed and infectivity titers were determined by a colorimetric plaque assay (**Lower panel**). The GDE-treated PVC matrices and controls were treated as described in (**A**). At the indicated time points, the viral inoculum was removed and replaced with a fresh inoculum on the same matrix. The infectivity was determined by a colorimetric plaque assay. Values are expressed as means ± SEM from three independent experiments. Differences were considered statistically significant for *p* < 0.05 (* *p* < 0.05; ** *p* < 0.01; *** *p* < 0.001, unpaired *t*-test, for comparison of GDE or DEHTP vs. CTRL samples).

To further validate our findings, we extended our investigation to other viruses, specifically HAdV, which served as a prototype of nonenveloped viruses, as well as HCoV-OC43, a closely related surrogate for human β-coronavirus SARS-CoV-2 and considered an example of RNA virus. The results showed that while the GDE-based matrices effectively inactivated HCoV-OC43, they did not reduce the infectivity of HAdV, suggesting that their antiviral properties may be due to the binding and disruption of the lipid envelope (Figure 4). These findings are in good agreement with previous studies demonstrating that free fatty acids are ineffective against nonenveloped viruses [36,37,38].

**Figure 5:**
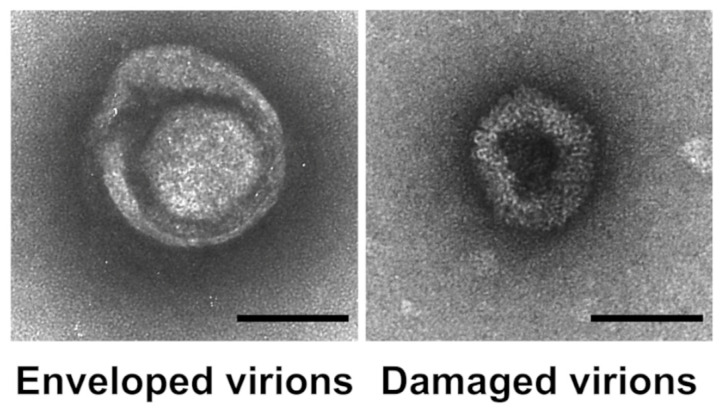
Exposure to GDE matrices disrupts the structural integrity of HSV-1 virions. HSV-1 was applied to GDE matrices. After 5 h, the virus was removed, and negatively stained preparations were observed using transmission electron microscopy (TEM) (**Left panel**). A representative micrograph of an intact virion showing typical herpesvirus morphology with a prominent outer envelope (**Right panel**). A representative micrograph of a virion with altered morphology and a damaged external envelope. Bar = 100 nm.

To confirm our hypothesis and examine the potential perturbation of enveloped virus membranes, we used TEM to visualize the morphology of HSV-1 virions after exposure to GDE matrices. We noticed distinct morphological differences in the HSV-1 exposed to GDE-treated matrices compared to those exposed to DEHTP. Besides the typical herpesvirus morphology, featuring an icosahedral structure surrounded by an external envelope, there were visibly altered virus particles upon exposure to the GDE matrices. These altered viruses appeared as deformed particles, probably due to damage to the viral structure and consequent leakage of the envelope, which allowed the stain to penetrate the particles. These data suggest that GDE interferes with the surface structure and integrity of HSV-1 virions (Figure 5).

Taken together, these results indicate that GDE matrices decrease the amount of viable enveloped viruses in a time- and dose-dependent manner by reducing the infectivity of virions. These findings thus imply that GDE matrices may represent a viable and effective solution for treating surfaces to curb the transmission of viruses via indirect surface exposure to infectious fluids.

### 3.6. Antibacterial Activity of GDE Matrices

**Figure 6:**
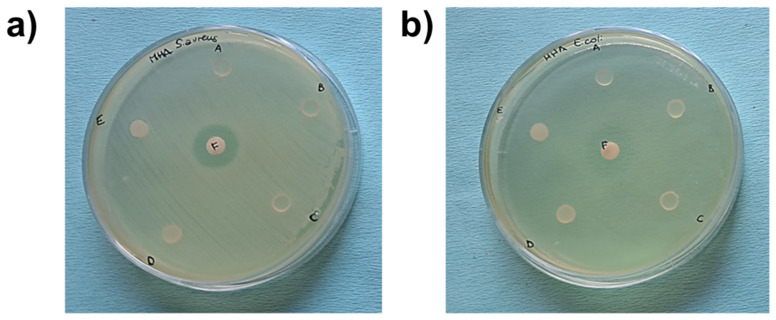
Photographic images of microbial inhibition zones against *S. aureus* (**a**) and *E. coli* (**b**) using DEHTP 50% (labeled as A), GDE 50% (B), GDE 36% (C), GDE 23% (D), and GDE 10% (E). Gentamicin was used as a positive control of the antimicrobial test (F). An inhibition halo was observed only around the gentamicin disk.

The antimicrobial activity of GDE matrices against Gram-positive *S. aureus* and Gram-negative *E. coli* was determined using the inhibition halo test. Photographic images of the microbial inhibition zones are shown in Figure 6.

**Figure 7:**
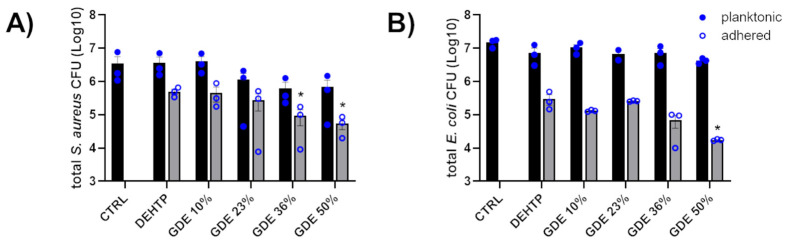
Antibacterial activity of GDE matrices against *S. aureus* (**A**) and *E. coli* (**B**). Sterile PVC-based disks plasticized with increasing concentrations of GDE (10%, 23%, 36%, and 50%), and DEHTP used as a control, were coated with bacterial suspensions of *S. aureus* or *E. coli* (1 × 10^4^ CFU/mL) and incubated at 37 °C for 5 h. Controls (CTRL), consisting of bacteria in the same inoculum size incubated in MHB with no material, were also set up. The total bacteria count, inclusive of adhered bacteria and planktonic forms, was quantified by CFU/mL count on agar plates. The graph displays the mean ± SEM from three independent experiments (* indicates statistical significance vs. DEHTP, *p* < 0.05; unpaired *t*-test).

The broad-spectrum antibiotic gentamicin was used as a positive control. The GDE matrices did not exhibit inhibitory activity toward the microorganisms studied, indicating that the matrices did not release any antibacterial compounds. This observation was further supported by counting the number of planktonic bacteria in contact with the PVC-based disks plasticized with increasing concentrations of GDE (Figure 7, black bars). Conversely, we observed a significative decrease in the adhered *S. aureus* when exposed to GDE 36% and GDE 50%, as well as a reduction in *E. coli* adherence with GDE 50%, compared to the control DEHTP. Specifically, the number of adhered *staphylococci* decreased from approximately 5 × 10^5^ CFU on DEHTP to about 9.4 × 10^4^ CFU (a reduction rate of about 81% compared to DEHTP) and 5.5 × 10^4^ CFU (a reduction rate of about 89% compared to DEHTP) on the GDE 36% and GDE 50% matrices, respectively. Similarly, *E. coli* adhered to the control matrix and GDE 50% with values of about 3 × 10^5^ CFU and 1.7 × 10^4^, respectively, which meant a reduction rate of about 94% compared to DEHTP (Figure 7, grey bars).

## 4. Discussion

In this study, we have successfully synthesized a new fatty acid epoxy-ester, named GDE, obtained from the esterification of soybean fatty acids with glycerol formal, followed by epoxidation of the double bonds with performic acid through conventional methods [39,40]. Our research demonstrates the viability of using GDE as a primary plasticizer for PVC materials. The resulting samples showed higher thermal stability compared to conventional petrochemical-based plasticizers, such as DINP, DINCH, DEHTP, and alternative bio-based plasticizers like P-401 and G-SNS. These results were further corroborated by the CR assay, revealing that GDE exhibited no discernible color change even after exposure to temperatures exceeding 180 °C for over 5 h, indicating the absence of significant degradation. ESBO-based plasticizers have been developed in recent years [41]. However, limitations such as low compatibility with PVC, poor plasticizing efficiency, and significant migration have restricted their broader application [42]. GDE effectively overcomes the poor plasticizing capacity of ESBO, thanks to its unique structural and electronic characteristics. We originally assumed that the increased polarity due to the glycerol formal group would enhance its compatibility with the PVC matrix, thus minimizing the plasticizer migration and consequent exudation. Indeed, our findings indicate that GDE can be incorporated up to 60 phr without any compatibility issues.

Analysis of the performance data, particularly the tensile strength and Shore A hardness, clearly indicates that GDE stands out as a highly efficient plasticizer. Its mechanical performance matches that of phthalates, yet it boasts exceptional thermal stability compared to petrochemical benchmarks. This property is particularly evident at 40 phr, while at 60 phr, the effect is similar, albeit less pronounced. Regarding the strain at break values, GDE displayed a better performance than DINP. Finally, the analysis of the tanδ curves for the 40 phr formulations highlighted the increased flexibility of GDE compared to DINP, DINCH, and DEHTP. Among the vegetable oil-based plasticizers, the GDE and G-SNS formulations were characterized by similar Tg values, while P-401 was not as effective in lowering the Tg of the blends, despite being structurally analogous to GDE. Currently, we can only offer preliminary findings, albeit encouraging ones, concerning GDE’s migration properties. The volatility measurements involved determining the mass loss of GDE over 7 days at 100 °C, utilizing a standard UL-type air exchange stove. In this evaluation, GDE displayed a performance similar to the reference plasticizer DINP. We acknowledge the importance of this matter and intend to address it in a more focused follow-up study.

Mounting evidence that contaminated surfaces greatly contribute to the spread of microorganisms [43,44,45] has increased the demand for flexible PVC products, such as medical disposables, flooring, and wallpaper, endowed with antimicrobial properties. Our results demonstrate that GDE-plasticized PVC exerts virucidal activity against enveloped viruses, including HSV-1 and HCoV-OC43. The effectiveness of the viral inactivation increases with higher concentrations of GDE and longer exposure, achieving up to 99.99% inactivation within 5 h at a minimum GDE concentration of 36%. The lack of effectiveness against nonenveloped viruses implies that GDE targets the viral envelope, which is composed of a phospholipid bilayer. This hypothesis is supported by the leakage of viral envelopes and disintegration of cell membranes observed by TEM analysis.

Some free fatty acids derived from milk and vegetable oils are known to have potent antiviral and/or antibacterial properties [46]. The efficacy of these fatty acids appears to strictly rely on the length of their hydrophobic moieties. Both medium-chain saturated (C8 to C14) and long-chain unsaturated fatty acids (C18–C20 and above) have shown significant activity against enveloped viruses. Thus, the blend of fatty acids in GDE, derived from soybean oil and specifically selected for their hydrocarbon chain comprised between C12 and C22, likely plays a significant role in its antiviral activity against enveloped virus.

Testing the practical applications of GDE, we found that its integration as a plasticizer in PVC led to the considerable antiviral activity of the material. Furthermore, reusability experiments involving GDE PVC demonstrated that it retained its antiviral capacity even after repeated exposures to the virus. Since limited data on the antimicrobial activity of PVC polymer blends are available, and we also evaluated GDE’s bactericidal and antiadhesive activities. Abdelghany AM et al. [47] showed that PVC with added polyvinyl acetate (PVAc) has some antibacterial activity toward only Gram-positive bacteria. In our experiments, when *S. aureus* and E. coli were incubated in suspensions with GDE PVC matrices, no reduction in their viable counts was observed, indicating that GDE, as a component of the PVC matrices, does not display bactericidal/bacteriostatic properties against planktonic cells. This finding also suggests that the GDE fatty acid, once immobilized on PVC, does not leach out into the surrounding environment. This inference was supported by the agar diffusion tests, which failed to detect any zones of bacterial growth inhibition around the GDE PVC matrices, further confirming the absence of leaching bactericidal agents from the material. However, we observed a significant reduction in the number of S. *aureus* adhered to the PVC with 36% and 50% GDE compared to the DEHTP control, whereas a significant decrease in *E. coli* adherence was only evident on 50% GDE PVC. We hypothesize that this reduction might primarily be due to a contact-active mechanism, even though a non-lethal inhibition of adhesion without killing cannot be ruled out, warranting further investigation. In this regard, previous studies have shown that tannin-coated surfaces can diminish Gram-positive bacteria adhesion due to blocking bacterium-substrate inhibition [48], and surfaces chemically modified with synthetic polymers can repel microorganisms without necessarily killing them [49,50,51].

Even though the antibacterial activity of GDE was significant, it was less pronounced than its virucidal activity against enveloped viruses. Intriguingly, a relationship between the free fatty acid structure and antibacterial activity remains quite controversial in the literature [52,53]. For instance, lauric acid (C12), a medium-chain aliphatic fatty acid, is known for its antibacterial properties, which are enhanced when it is esterified with glycerol to form monolaurin. Nevertheless, it is important to point out that most fatty acids do not exhibit antimicrobial activity at moderate concentrations and that their combined effects can lead to synergistic or antagonistic effects depending on the component ratio [54,55,56]. In line with our results, Diez-Pascual AM et al. found that nanocomposites with neat ESO displayed some antimicrobial activities that were more effective against Gram-positive than Gram-negative bacteria [57,58].

In summary, PVC plasticized with GDE not only shows promising antimicrobial properties but also retains favorable mechanical and thermal characteristics. This combination makes it a compelling alternative to conventional petrochemical and vegetable oil-based plasticizers for PVC. Lastly, its application could be highly beneficial in environments where the risk of viral disease transmission through physical contact is high.

## 5. Conclusions

We have synthesized GDE, a novel plasticizer derived from soybean oil and glycerol formal vegetable oils, and incorporated it into PVC. Our findings demonstrate the antimicrobial properties of GDE-based materials, which are particularly effective in killing enveloped viruses, such as HSV-1 and HCoV-OC43. TEM analysis indicates that GDE disrupts the viral structure, potentially leading to envelope leakage. In contrast, the impact of GDE on nonenveloped viruses like HAdV is negligible. Importantly, GDE also displays antibacterial activity against two common pathogenic bacteria, *S. aureus* (Gram-positive) and *E. coli* (Gram-negative). Given these properties, GDE emerges as a promising antimicrobial compound that may be used in both community and healthcare settings to prevent infection spread. Future investigations will focus on an in-depth characterization of the material, encompassing DMTA analysis at elevated temperatures, UV–Vis assessments of PVC foils, and dedicated cytotoxicity assays. These findings will allow us a thorough understanding of the safety and suitability of GDE for medical devices.

## 6. Patents

The authors declare the following competing financial interests: Giuseppe Chiaradia is a partner in the company Fluos s.a.s. Busto Arsizio, Varese, Italy; Patent No. PCT/EP2014/074208.

## Figures and Tables

**Table 1:** List of the materials used in this study.

Product	Manufacturer	IUPAC Name	CAS Number
Plasticizer			
Fatty acid epoxy-ester of glycerol formal (GDE)	Fluos (Busto Arsizio, Italy)	Fatty acids, C16-22, epoxidized, glycerol formal esters	2764604-30-8
Di-isononyl phthalate (DINP)	BASF (Ludwigshafen, Germany)	Diisononylphthalate	28553-12-0
Di-isononyl-1,2-cyclohexanoate (DINCH)	ex-BASF (Mortara, Italy)	Bis(7-methyloctyl) cyclohexane-1,2-dicarboxylate	166412-78-8
Diethyl hexyl terephthalate (DEHTP)	Sigma-Aldrich (Zwijndrecht, The Netherland)	Di-(2-ethylhexyl) terephthalate	6422-86-2
Grindsted soft-N-safe (G-SNS)	IFF (Grindsted, Denmark)	1,3-bis(acetyloxy)propan-2-yl 12-(carboxyoxy)octadecanoate; 2,3-bis(acetyloxy)propyl 12-(acetyloxy)octadecanoate	736150-63-3
P-401	Traquisa (Barcelona, Spain)	Fatty acids, 2-ethylhexyl ester, epoxidized	95370-96-0
PVC *			
P 1340 K70 Ultra	Vestolit Gmbh (Marl, Germany)	Poly(1-chloroethylene)	9002-86-2
Stabilizer			
Ba-Zn stabilizer	Reagens (San Giorgio di Piano, Italy)	N/A	N/A
Epoxidized soybean oil (ESBO)	Emery Oleochemicals (Loxstedt, Denmark)	Soybean oil, epoxidized	8013-07-8

Ba-Zn = barium-zinc. N/A = not available. * K value: 70; Mw = ~83,000 Da; Mn = ~40,000 Da [23].

**Table 2:** PVC formulations’ components in grams.

Material	Type	A (40 phr) *	B (60 phr) *
PVC	Vestolit P 1340	80	70
Plasticizer	GDE, DINP, DINCH, DEHTP, G-SNS, P-401	32	42
Primary stabilizer	Ba-Zn stabilizer	1.6	1.4
Secondary stabilizer	ESBO	2.4	2.1
	Total	116	115.5

* formulations A and B based on either 40 or 60 phr plasticizer content; phr = parts per hundred resin.

**Table 3:** Comparative summary of the physical and chemical properties of GDE and the control samples. The italicized values were product specifications provided by the manufacturers.

Property	Description	Unit	GDE	DINP	DINCH	DEHTP	P-401	G-SNS
Ester content	GC	Area (%)	95.7	*99.6*	*99.5*	*99.9*	98.4	97.0
Acid value	Titration	mg KOH/g	1.13	*0.04*	*0.05*	*0.03*	*0.47*	*3.00*
Water content	Karl-Fischer	Weight (%)	0.04	*0.05*	*0.10*	*0.08*	0.02	*0.50*
Viscosity	20 °C	mPa s	113.8	*72–82*	*44–60*	*77.6*	*46*	123.2
Volatiles	TGA	Weight (%)	−0.12	0.22	−0.16	−0.12	0.57	−0.05
Ash content	TGA	Weight (%)	0.1	0.23	0.09	−0.05	0.13	−0.13
Color	APHA	-	338	*30*	*40*	*10*	99	100

**Table 4:** Summary of the effect of the different plasticizers on the thermal properties measured as the long-term heat stability in minutes.

Plasticizer	phr	Long-Term Heat Stability (min)
DINP	40	33
DINCH	40	36
DEHTP	40	37
P-401	40	>67
G-SNS	40	41
GDE	40	>67
DINP	60	35
DINCH	60	45
DEHTP	60	50
P-401	60	>67
G-SNS	60	48
GDE	60	>67

**Table 5:** Results of the Congo red test of the 40 phr PVC compounds.

PVC Compounds	Thermal Stability Time (min) Mean
DINP	85.5
GDE	>330
P-401	>330

**Table 6:** Tensile and mechanical properties of the different plasticized PVC matrices. Mean ± SD is shown (five measurements per sample).

Plasticizer	phr	E-Modulus (Mpa)	F-max (Mpa)	Strain at Fracture (%)	Shore A Hardness
DINP	40	31 ± 4	25.6 ± 1.3	281 ± 7	34 ± 1
DINCH	40	55 ± 4	22.0 ± 5.3	196 ± 73	52 ± 2
DEHTP	40	48 ± 4	24.4 ± 2.2	147 ± 20	42 ± 0
P-401	40	35 ± 2	18.2 ± 3.6	152 ± 46	26 ± 1
G-SNS	40	23 ± 1	18.2 ± 3.8	143 ± 42	29 ± 1
GDE	40	15 ± 2	22.9 ± 3.1	220 ± 40	28 ± 1
DINP	60	7 ± 1	13.2 ± 4.6	203 ± 82	28 ± 1
DINCH	60	8 ± 0	12.1 ± 0.9	179 ± 25	29 ± 1
DEHTP	60	7 ± 0	16.9 ± 1.3	267 ± 24	26 ± 1
P-401	60	7 ± 1	12.9 ± 1.3	241 ± 45	25 ± 1
G-SNS	60	6 ± 0	15.9 ± 2.8	282 ± 63	21 ± 1
GDE	60	5 ± 0	15.8 ± 2.2	338 ± 43	26 ± 1

## Data Availability

The raw data supporting the conclusions of this article will be made available by the authors upon request.

## Supplementary Materials

The following supporting information can be downloaded at https://www.mdpi.com/article/10.3390/polym16081046/s1. Supplementary Figures with the analysis of the ester content in the GDE plasticizer by GC-MS (Supplementary Figure S1). Supplementary Figure reporting the physical appearance of the roll-milled PVC sheets (Supplementary Figure S2). Supplementary Figure for the comparison of the tanδ curves of the 40 phr plasticized PVC compounds (Supplementary Figure S3).
