# Supplementary material for: Antimicrobial Efficacy of a Vegetable Oil Plasticizer in PVC Matrices
Source: Polymers (Basel). 2024 Apr 10;16(8):1046. doi: 10.3390/polym16081046 (PMC11054656; doi:10.3390/polym16081046)
Supplement: Supplementary file 1 [file polymers-16-01046-s001.zip › polymers-2920455-supplementary.pdf]

polymers

MDPI

Supporting Information

Article

Antimicrobial efficacy of a vegetable oil plasticizer in PVC matrices

Greta Bajetto[1,2#], Sara Scutera[1#], Francesca Menotti[1], Giuliana Banche[1], Giuseppe Chiaradia[3], Caterina Turesso[3], Marco De Andrea[1,2], Marta Vallino[4], Daan S. Van Es[5], Matteo Biolatti[1*], Valentina Dell'Oste[1], Tiziana Musso[1]

1 Department of Public Health and Pediatric Sciences, University of Turin, Turin, Italy
greta.bajetto@unito.it (G.Baj.); sara.scutera@unito.it (S.S); francesca.menotti@unito.it (F.M.); giuliana.banche@unito.it (G.Ban.); marco.deandrea@unito.it (M.D.A.); matteo.biolatti@unito.it (M.B.); valentina.delloste@unito.it (V.D.O.); tiziana.musso@unito.it (T.M.)

2 Center for Translational Research on Autoimmune and Allergic Disease-CAAD, Novara, Italy

3 Fluos Sas, Busto Arsizio, Varese, Italy
chiaradia@fluos.it (G.C.); turesso@fluos.it (C.T.)

4 Institute for Sustainable Plant Protection, CNR, Turin, Italy
marta.vallino@ipsp.cnr.it (M.V.)

5 Wageningen Food & Biobased Research, Wageningen, The Netherlands.
daan.vanes@wur.nl (D.S.V.E.)

# contributed equally
* Correspondence: matteo.biolatti@unito.it; Tel.: +39-011-6705635

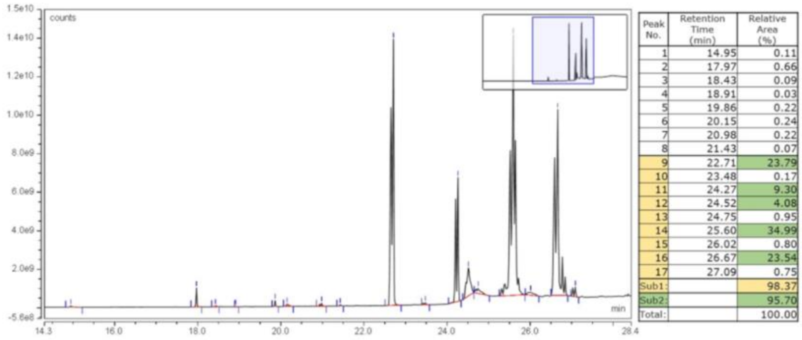

Peak No.	Retention Time (min)	Relative Area (%)
1	14.95	0.11
2	17.97	0.66
3	18.43	0.09
4	18.91	0.03
5	19.86	0.22
6	20.15	0.24
7	20.98	0.22
8	21.43	0.07
9	22.71	23.79
10	23.48	0.17
11	24.27	9.30
12	24.52	4.08
13	24.75	0.95
14	25.60	34.99
15	26.02	0.80
16	26.67	23.54
17	27.09	0.75
Sub1:		98.37
Sub2:		95.70
Total:		100.00

Supplementary Figure. S1. Analysis of ester content in GDE plasticizer by GC-MS. The ester content is based on peak areas. Sub 1 refers to the sum of main peaks (numbers 9, 11, 12, 14, 16) and peaks between main peaks (numbers 10, 13, 15, 17), while Sub2 refers to the main peaks only.

(a) and 60 (b) phr.

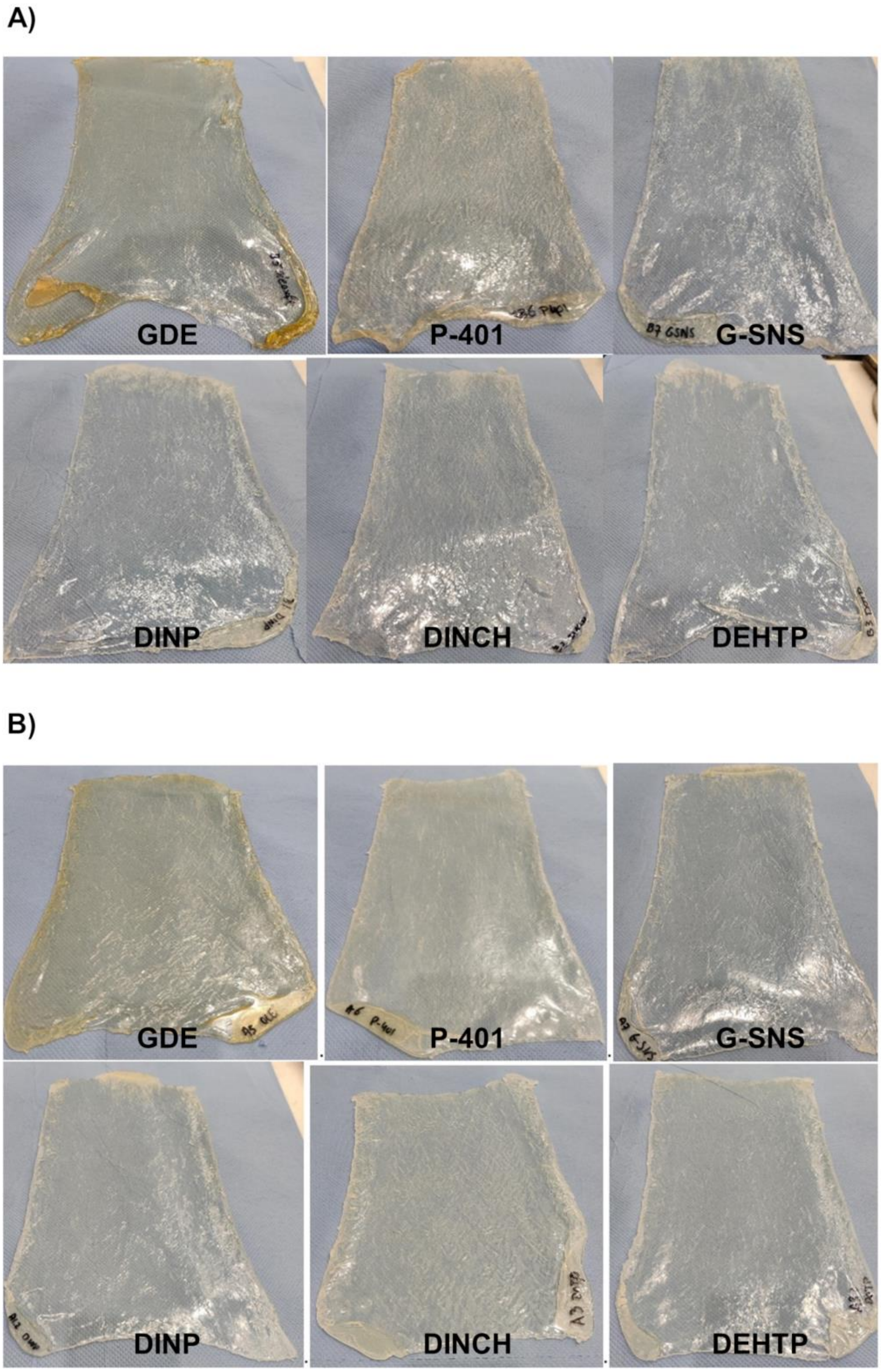

Supplementary Figure. S2. Physical appearance of roll-milled PVC sheets at concentrations of 40 (a) and 60 (b) phr.

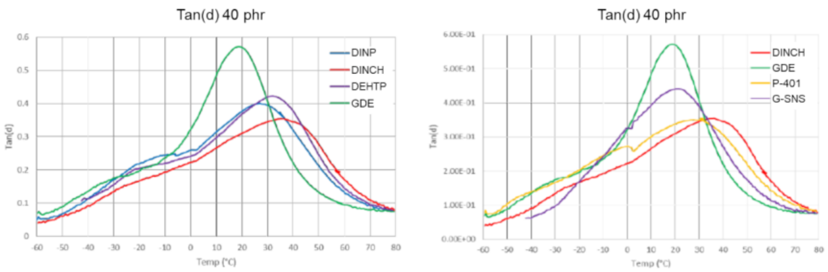

Supplementary Figure. S3. Comparison of the tanδ curves of 40 phr plasticized PVC compounds.
